# Editorial: Highlights in diabetes self-management 2021/22

**DOI:** 10.3389/fcdhc.2023.1116879

**Published:** 2023-01-13

**Authors:** Roberta Lamptey, Emma Berry, Norbert Hermanns, Frank Snoek

**Affiliations:** ^1^ Family Medicine Department, Korle Bu Teaching Hospital, Accra, Ghana; ^2^ Jullius centre, University medical centre Utrecht, Utrecht, Netherlands; ^3^ Department of Community Health, University of Ghana Medical School, University of Ghana, Accra, Ghana; ^4^ School of Psychology, Queen’s University Belfast, Northern Ireland, Belfast, United Kingdom; ^5^ Research Institute of the Diabetes Acdemy Mergentheim (FIDAM), Bad, Mergentheim, Germany; ^6^ Department of Medical Psychology, Amsterdam University Medical Center (UMC), Vrije Universiteit, Amsterdam, Netherlands

**Keywords:** diabetes, self-management, education, self-care - diabetes knowledge, self-care (MeSH)

Self-management is arguably the cornerstone and the most challenging aspect of diabetes care for people living with diabetes, irrespective of the type of diabetes. Self-management encompasses the routines necessary for managing glycaemic levels and includes; glucose monitoring, portioning meals/snacks, meal timing, physical activity, taking medication, healthy coping, and foot care. Self-care can be demanding for people living with diabetes, as it requires cognitive, emotional, and behavioural effort on a daily basis. Sustaining self-care routines over a lifetime is burdensome; however, this enduring effort is important in supporting long-term health and wellbeing.

People living with diabetes should therefore have access to continuous diabetes self-management support. Unfortunately, this is often not the case, particularly in low-income countries. Even in well-resourced settings, access to self-management education and support is often limited and not provided on a structural basis. Clearly, person-centred self-management education and support deserves more attention, both in youth and adults with diabetes.

Evidence from papers in this collection are summarised in the sections below.

## Playful communication and care: Exploring child-centred care of young children with type 1 diabetes through the framework of zone of proximal development

Using qualitative methods specifically observation and interviews DeCosta et al examined established care practices which address the mental and social needs of young children living with type 1 diabetes (T1DM), by interviewing professionals from specialist centres in Denmark. Half of the professionals interviewed had up to 19 years of experience in paediatric endocrinology. Although their findings cannot be generalised they provide rare insights into approaches to diabetes self-management in children: very young children.

Attention to immediate needs, child centeredness and participation, and communication style were themes emerging from their study. Delivering diabetes care using these strategies facilitated the process of drawing these young children into their self-care.

### Attention to immediate needs

DeCosta et al. report a paradigm shift from the biomedical model to the biopsychosocial model, in providing care for children with T1DM. Deliberate effort now goes into improving a child’s experience in hospital at the time of diagnosis. Consistent messaging from the same team of healthcare providers and predictability helps a child living with T1DM to build relationships and helps to create a safe environment for the child. Learning, including learning about diabetes and self-care happens when a child feels secure. At the time of diagnosis, the immediate needs of children living with T1DM should be and can be met by limiting change, limiting pain, and enhancing predictability.

### Child centeredness

By removing the focus from diabetes to the child as a whole person and what matters to them the providers invest in the ability of the child to share diabetes self-care related challenges with clinicians in the future. Connecting to the child on matters unrelated to diabetes management provides a channel for effective diabetes self-management education and support.

### Child participation

During consultations, clinicians prevent ‘sugars’ from getting in the way. Abstract numbers are complex for children and prevent them from getting involved in their care. Clinicians ensured that consultations were pleasant experiences by using several techniques to limit pain and fear. Role play improved child involvement; but more importantly helped them recover from unpleasant experiences.

### Playful communication

Understanding the child’s world and using positive feedback and gifts made it possible to earn children’s corporation for unpleasant procedures.

## Medication intake, perceived barriers, and their correlates among adults with type 1 and type 2 diabetes: Results from diabetes MILES – The Netherlands

Factors associated with self-reported medication taking may be modified to improve medication taking. Hogervorst et al. reported that depression and diabetes-related distress were negatively associated with medication taking in both T1DM and T2DM. Suggesting that in adults living with diabetes self-care is influenced by mental health.

Furthermore, medication taking was associated with lower HbA1c values for all sub-types of diabetes. Adults living with diabetes on insulin therapy irrespective of sub-type of diabetes were shown to be taking their medication more often than adults living with diabetes who were not on insulin. However, the effect sizes were small.

Importantly, among adults living with T1DM, fewer events of severe hypoglycaemia was associated with less frequent medication taking and more perceived barriers to taking medication, after adjusting for multiple confounders. This finding underlines the complexity of self-management in T1DM. In adults living with T2DM shorter duration of diabetes and mental health challenges remained positively associated with not taking medication and perceived barriers to medication taking.

These findings underscore the need for clinicians to be alert to characteristics in adults living with T1DM and T2DM, which may pre-dispose persons living with diabetes to depression, not taking medication, and increased perceived barriers to medication taking. These factors should also addressed as part of modern individualised diabetes self-management education.

The study population in this study were a non-representative sample of Dutch adults with self-reported diabetes participating in an online cross-sectional study. The generalisability of these findings may therefore be limited.

## Empowered by intertwined theory and practice – experiences from a diabetes sports camp for physically active adults with type 1 diabetes

Self-management education does not always translate into improved self-care behaviours for multiple reasons. Wide variations in glycaemic levels can frustrate self-management efforts of physically active people. These challenges may be mitigated by empowering through education. Mattsson et al. employ qualitative techniques (telephone interviews) to study how new learnings become habits. The study population comprised of 15 people living with T1DM for a mean of 15 years, who participated in a 3-day diabetes sports camp. Self-reported increases in diabetes specific knowledge was observed after immersion in a sports camp. The presence of peers, and engagement with multi-disciplinary teams, practical sessions, timely individualised feedback including technology-assisted feedback, seemed to have facilitated the process of behaviour change.

## Making diabetes care fit—are we making progress?

This perspective piece by Ruissen et al. shed light on the overarching principle that diabetes care needs to be tailor-made. Unfortunately, one-size simply cannot fit all. It is not only impossible but also impractical to deliver tailor made diabetes self-management education and support without involving the patient and their support persons. Patient centeredness must be the guiding principle for all diabetes related encounters.

The studies summarised above are reflective of the complexity of self-management in diabetes. They provide an insight into the determinants of self-management as well as potentially useful interventions to support self-management in a way that centres on the holistic needs of persons living with diabetes.

## Author contributions

RL prepared the first draft. All authors made modifications and approved the final version.

